# Assessment of mechanical properties of isolated bovine intervertebral discs from multi-parametric magnetic resonance imaging

**DOI:** 10.1186/1471-2474-13-195

**Published:** 2012-10-12

**Authors:** Maximilien Recuerda, Delphine Périé, Guillaume Gilbert, Gilles Beaudoin

**Affiliations:** 1 Mechanical Engineering Department, Ecole Polytechnique de Montréal, Montréal, QC, Canada; 2 Research Center, CHU Sainte-Justine, Université de Montréal, Montréal, QC, Canada; 3 Philips Healthcare, Montréal, QC, Canada; 4 CHUM Saint-Luc, Université de Montréal, Montréal, QC, Canada

**Keywords:** Intervertebral disc, Compression tests, Mechanical properties, Multi-parametric MRI, Multiple linear regressions

## Abstract

**Background:**

The treatment planning of spine pathologies requires information on the rigidity and permeability of the intervertebral discs (IVDs). Magnetic resonance imaging (MRI) offers great potential as a sensitive and non-invasive technique for describing the mechanical properties of IVDs. However, the literature reported small correlation coefficients between mechanical properties and MRI parameters. Our hypothesis is that the compressive modulus and the permeability of the IVD can be predicted by a linear combination of MRI parameters.

**Methods:**

Sixty IVDs were harvested from bovine tails, and randomly separated in four groups (*in-situ*, *digested-6h*, *digested-18h*, *digested-24h*). Multi-parametric MRI acquisitions were used to quantify the relaxation times T1 and T2, the magnetization transfer ratio MTR, the apparent diffusion coefficient ADC and the fractional anisotropy FA. Unconfined compression, confined compression and direct permeability measurements were performed to quantify the compressive moduli and the hydraulic permeabilities. Differences between groups were evaluated from a one way ANOVA. Multi linear regressions were performed between dependent mechanical properties and independent MRI parameters to verify our hypothesis. A principal component analysis was used to convert the set of possibly correlated variables into a set of linearly uncorrelated variables. Agglomerative Hierarchical Clustering was performed on the 3 principal components.

**Results:**

Multilinear regressions showed that 45 to 80% of the Young’s modulus E, the aggregate modulus in absence of deformation H_A0_, the radial permeability k_r_ and the axial permeability in absence of deformation k_0_ can be explained by the MRI parameters within both the nucleus pulposus and the annulus pulposus. The principal component analysis reduced our variables to two principal components with a cumulative variability of 52-65%, which increased to 70-82% when considering the third principal component. The dendograms showed a natural division into four clusters for the nucleus pulposus and into three or four clusters for the annulus fibrosus.

**Conclusions:**

The compressive moduli and the permeabilities of isolated IVDs can be assessed mostly by MT and diffusion sequences. However, the relationships have to be improved with the inclusion of MRI parameters more sensitive to IVD degeneration. Before the use of this technique to quantify the mechanical properties of IVDs in vivo on patients suffering from various diseases, the relationships have to be defined for each degeneration state of the tissue that mimics the pathology. Our MRI protocol associated to principal component analysis and agglomerative hierarchical clustering are promising tools to classify the degenerated intervertebral discs and further find biomarkers and predictive factors of the evolution of the pathologies.

## Background

The planning of the treatment of spine pathologies requires information on the rigidity of the tissues. The surgeons use side-bending radiographs to estimate the rigidity of the spine, but the results vary with the muscular effort made by the patient [1,2]. A fulcrum bending test was developed to more accurately reflect the spine flexibility [3] as well as a suspension test [4]. Reverse methods including a finite element model of the spine have been developed but the segmentation process of the radiographs and the inversion of the reverse problem are computationally challenging. Consequently, these methods are not clinically used on a regular basis, and the information remains global.

The intervertebral disc (IVD) plays an important role in the mobility of the vertebral segments. As IVDs degenerate, the nucleus pulposus becomes more consolidated and fibrous, and is less clearly demarcated from the annulus fibrosus in which focal defects appear and there is a decrease in the number of layers [5,6]. The mechanical behaviours of the IVD matrix and their changes with degeneration have been widely investigated on nonviable tissue in vitro, with reported loss of disc height, fluid pressurization and hydration, and altered compressive modulus, shear modulus, or permeability [7-11]. Thus it is important to be able to quantify the compressive modulus of the IVD to assess the spine rigidity. Moreover, the IVD is an avascular tissue, except in the outermost annulus fibrosus, and its nutrition is achieved by diffusion from the vertebral endplates [12]. A reduced permeability of the disc will decrease the nutrition of the inner AF and nucleus pulposus, and increases their degeneration. Thus it is important to be able to quantify the permeability of the IVD. Moreover, the permeability may vary between the endplates border of the healthy disc, where there is the diffusion of the nutriments and the periphery of the annulus fibrosus. However, we only know the changes in the zero-strain permeability from the nucleus pulposus (0.68±0.09*10^-15^m^4^N^-1^s^-1^) to the annulus fibrosus (0.24±0.19*10^-15^m^4^N^-1^s^-1^) as computed from confined compression tests and non linear biphasic models, with no differentiation between inner and outer annulus fibrosus [10,13].

Multi-parametric MRI has been investigated as an early diagnostic tool of IVD degeneration by correlating the MRI parameters to the IVD degeneration. IVD water, proteoglycan and collagen contents were found to be correlated to the longitudinal relaxation time (T1), the transverse relaxation time (T2), the time constant of the exponential decay of magnetization during a spin-lock radiofrequency pulse (T1ρ), the magnetization transfer ratio (MTR) and the diffusion [14-17]. T1 and T2 decreased when the Thompson grade increased in the nucleus pulposus [16,18,19]. Magnetization transfer (MT) sequence was sensitive to Thompson grades in in-vivo studies [18] and slightly to Pfirrmann grades [20]. Furthermore changes in the extracellular matrix structure and fiber organization altered the MTR [16]. The apparent diffusion coefficient (ADC) decreased with increasing Thompson grades and also with increasing loading of the IVD [19,21]. Antoniou et al. [17] showed that the ADC in the nucleus pulposus decreased with a decrease in proteoglycan and water contents. These decreased ADC values reflected the lost integrity of the intervertebral [22].

As a result of these correlations between the MRI parameters and the biochemical properties and between the biochemical properties and the mechanical properties [9,23], multi-parametric MRI was used to estimate the mechanical properties of IVD tissues. The hydraulic permeability and T1 were found to be correlated in caudal bovine nucleus pulposus [11]. Weak linear correlation was found between T1ρ and the osmotic pressure of the human nucleus pulposus [24]. On bovine discs, strong relationships were found between permeability and MRI parameters including T1ρ in the nucleus pulposus and correlations were found between axial permeability and T1ρ in the annulus fibrosus and between compressive modulus and T1ρ in the nucleus pulposus [25]. T2 and permeability were correlated in bovine nucleus pulposus and the only mechanical property associated to the MR diffusion was the permeability, and a trend was found between ADC and H_A0_[26]. MRI scaling using Gibson’s scale on T2-weighted MRI images was able to categorize the elastic modulus and the viscosity of the IVDs in two clearly distinct groups without overlaps according to degeneration [27]. MRI offers great potential as a sensitive and non-invasive technique for describing the alterations in mechanical properties of IVDs. However, the literature showed large standard deviations in the determination of the mechanical properties of the IVD and small coefficients of correlation between mechanical properties and MRI parameters. The assessment of the permeability value was usually done by mathematical model regression, but it can be estimated more accurately by direct measurement using a custom setup according to the Darcy theory [28-30].

Our hypothesis is that the compressive modulus and the hydraulic permeability of the IVD can be predicted by a combination of MRI parameters (T1, T2, MTR, ADC, fractional anisotropy FA). Our specific aim is to measure the mechanical properties, using confined and unconfined compression tests and direct permeability measurements, and the MRI parameters of isolated bovine IVDs and to investigate the relationships between these parameters. Bovine caudal IVDs from 6 months old animals are usually healthy. Thus, to validate the use of multi-parametric MRI to evaluate the mechanical properties of degenerated IVDs, we digested the bovine IVDs. Trypsin is known to decrease the Young’s modulus, increase the permeability and alter the structure of the disc [11,23].

## Methods

### Samples preparation

Bovine tails were obtained from a local slaughterhouse within 4 hours of death. The skin, muscles, ligaments and vertebrae pedicles were removed from each tail. Transverse cuts were performed along the cartilaginous endplates to isolate the two proximal discs. Sixty discs with a controlled 15mm thickness were randomly separated in four groups. The discs from the *in-situ* group (n=15) were wrapped in plastic to prevent dehydration. The discs from each of the 4 *digested* groups (n=15 per group) were digested in PBS solution with a specific concentration of trypsin from bovine pancreas (T8003 Sigma-Aldrich), during a varying amount of time at 37° with shaking. The duration of the digestion and the concentration of the trypsin are summarized in Table 1. Trypsin, a mammalian serine protease, catalyzes the hydrolysis of peptide bonds at the carboxyl side of lysyl and arginyl residues. Trypsin was chosen to induce general degradation of the disc structure and composition. A light platen of plastic was placed on the top of the disc to avoid bulging during the treatment. Their wet weights were measured before and after digestion to quantify the percentage of hydration.

**Table 1:** Trypsin digestion groups

**Group**	**Duration**	**Concentration [mg/ml]**
*Digested-6h*	6h	0.05
*Digested-18h*	18h	0.1
*Digested-24h*	24h	0.5

### MR imaging

**Figure 1:**
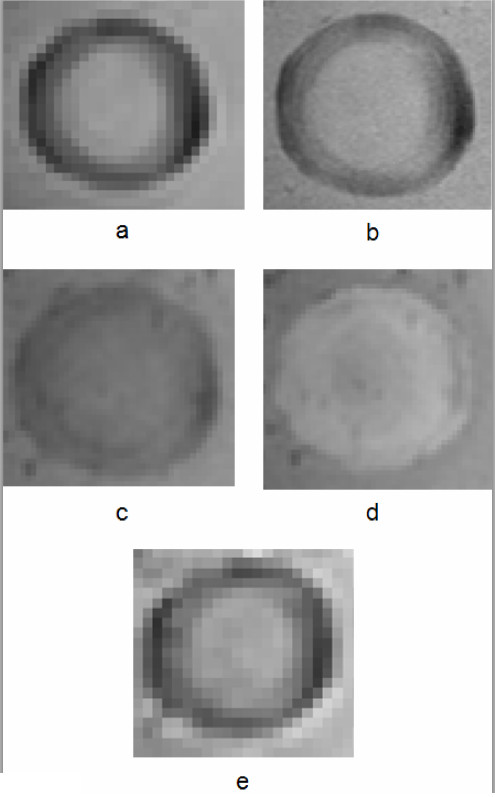
Example of T1 weighted image (a), T2 weighted image (b), MT image with the off-resonance pulse (c), MT image without the off-resonance pulse (d) and Diffusion weighted image (e) for an isolated IVD.

Immediately after digestion, samples were removed from the shaking water bath, and were gently blotted to remove absorbed water. Thickness and weight of discs from both groups (*digested* and *in-situ*) were measured just before the MRI acquisition. Discs were placed in chambers filled with PBS solution. A light platen was placed on the top to stabilize the discs during the MRI acquisition. The acquisition was performed using a 3T whole-body system (Philips Achieva X-Series). Images for the quantification of T1 (Figure 1-a) and T2 (Figure 1-b) were acquired using a multiple inversion recovery turbo spin-echo (TSE) sequence for T1 (TR/TE=2100/6.3ms, 15 inversions times (TI) from 50 to 1900ms) and a multi-echo turbo spin-echo (TSE) sequence for T2 (TR=2000ms, 10 TE every 15ms). T1 and T2 were extracted (Matlab, r2007 Mathworks, Natick, MA) from the signal intensity (SI) by fitting equations 1 and 2 [31].

(1) $$SI_{\text{TI}}=S_{0}\left\{1-\left(1-cos\left(\alpha \right)\right)E_{\text{TI}}\left(1-E_{TW^{f^{N}}}\left(1-\frac{E_{\text{TE}/2}}{2}\right)\right)\right\}$$

with $f\left(x\right)=1-E_{\text{TE}}x,\;E_{\text{TI}}=e^{-\frac{TI}{T1}},\;E_{\text{TW}}=e^{-\frac{TW}{T1}},\;E_{\text{TE}}=e^{-\frac{TE}{T1}}$ and N=8.

(2) $$SI_{\text{TE}}=SI_{\left(TE=0\right)}e^{-\text{TE}/T2}$$

Where SI is the signal intensity, α the refocusing angle =180º, TW the time in ms between the last refocusing pulse and the next inversion pulse, and N the number of refocusing pulses.

The MTR was obtained using two gradient echo sequences (TR/TE=83/3.8 ms, single off-resonance sinc-gauss pulse, 19.25 ms duration, 620 degrees effective flip angle), one with the off-resonance pulse applied at 1100Hz down to the free water proton resonance frequency (Ms, Figure 1-c) and the other one without it (Mo, Figure 1-d) [20]. MT ratio was calculated from equation 3 [32].

(3) $$MTR=\frac{M_{0}-M_{S}}{M_{0}}$$

The last sequence measured the ADC and FA using a spin-echo EPI diffusion-weighted sequence (TR/TE=2000/40 ms) with 15 non-collinear diffusion and a b value of 1000 s/mm^2^ (Figure 1-e). ADC and FA were calculated using equations 4, 5, 6 [33-35]:

(4) $$SI_{\left(b\right)}=SI_{\left(b=0\right)}e^{-b.D}$$

(5) $$ADC=\frac{\lambda_{1}+\lambda_{2}+\lambda_{3}}{3}$$

(6) $$F_{A}=\sqrt{\frac{3\left[{\left(\lambda_{1}-ADC\right)}^{2}+{\left(\lambda_{2}-ADC\right)}^{2}+{\left(\lambda_{3}-ADC\right)}^{2}\right]}{2\left[\lambda_{1}^{2}+\lambda_{2}^{2}+\lambda_{3}^{2}\right]}}$$

Where **b** is the diffusion encoding tensor, **D** the diffusion tensor and λ the eigenvalues of D.

Each MR image was semi-automatically segmented using Slice-O-Matic (Tomovision, Magog, Canada). A manual selection of 8 points on the exterior outline of the IVD and nucleus pulposus allowed the contour to be approximated by the Snake algorithm. A manual correction of each contour was then realized to improve their anterior and posterior extremities. The annulus fibrosus zone was obtained by the subtraction of the nucleus pulposus zone from the IVD zone. The repetition of the semi-automatic segmentation by a same operator and the skills of the operator did not influence the quality of the contour (p=0.8-1.0) while the instructions given prior to the segmentation influenced the quality of the contour (p<0.05).

### Mechanical testing

Just after the MRI acquisitions, all discs were frozen (−80°C) until the day of mechanical testing to avoid any additional enzyme degradation or dehydration of the tissue. It is recognized that freezing does not affect the determination of the mechanical properties of IVDs and it facilitates the preparation of the tissue [25,26]. Three mechanical tests were performed: unconfined and confined compression tests and direct permeability measurement. IVDs were punched with a 5mm diameter punch in both the nucleus pulposus and posterior annulus fibrosus regions. Each punched sample was divided into four slices of height 1.6±0.5mm, three slices being tested mechanically and one being reserved for biochemical measurements. The protocols for confined and unconfined compression tests of IVDs were based on previous studies in which we identified the initial testing conditions allowing the highest reproducibility of the measures. In unconfined compression, the protocol including an initial swelling, a 5% strain preload and a 5% strain ramp is the most relevant protocol to test the annulus fibrosus while the protocol with semi confined swelling and a 5% strain ramp is the most relevant protocol for the nucleus pulposus [36]. In confined compression, the best initial condition was the confined swelling followed by a 5% strain ramp [13].

#### Unconfined compression

The unconfined compression test was performed using the mechanical testing machine Mach-1 (Biomomentum, Montreal, QC, Canada). Before the test, tissues were bathed during 10 minutes in PBS in free condition for the annulus fibrosus or in semi-confined condition (allowing only radial displacement) for the nucleus pulposus. After the tissue sample was placed in the chamber for the unconfined compression test, the upper platen was lowered until a stable force of 8 × 10^-5^N for the annulus fibrosus or 6 × 10^-5^N for the nucleus pulposus was recorded, indicating that the platen was in contact with the top of the specimen with no deformation of the specimen. The thickness of the tissue sample was then deduced from the relative position of the upper platen to the bottom of the chamber. A preloading of 5% strain during 10min was applied to the annulus fibrosus. Five successive stress-relaxation ramps were applied using 5% strain increment. The relaxations were stopped when the slope of the curve reached a rate of 0.1g per min. The mechanical properties were computed (Matlab, r2007 Mathworks, Natick, MA) using a viscoelastic model [37] to evaluate the Young’s modulus or (E) and the viscocity (μ), and a linear biphasic poroviscoelastic mathematical model [38,39] to evaluate the radial permeability (k_r_), the Poisson’s ratio (ν), and the viscoelasticity (c). The Young’s modulus or modulus of elasticity can be used to predict the elongation or compression of an object as long as the stress is less than the yield strength of the material. Viscosity describes a fluid’s internal resistance to flow and may be thought of as a measure of fluid friction. The hydraulic permeability indicates the resistance to fluid flow through the intervertebral disc matrix. The Poisson’s ratio is the ratio of transverse contraction strain to longitudinal extension strain in the direction of stretching force. Viscoelasticity is the property of materials that exhibit both viscous and elastic characteristics when undergoing deformation, for which the relationship between stress and strain depends on time.

#### Confined compression

The confined compression test was performed with the same apparatus used for the unconfined compression test. A custom non porous acrylic chamber (5 millimeters diameter) was designed and manufactured. The protocol described in previous studies [10,13] was used. However, the relaxation period was stopped when the force slope was lower than 0.1g/min. The aggregate modulus H_A0_ and the axial hydraulic permeability k_0_ for zero-strain and their respective nonlinear coefficients β and M were computed from a non linear biphasic model [10,40-42]. The aggregate modulus is a measure of the stiffness of the tissue at equilibrium when all fluid flow has ceased. The higher the aggregate modulus, the less the tissue deforms under a given load.

#### Permeability measurement

**Figure 2:**
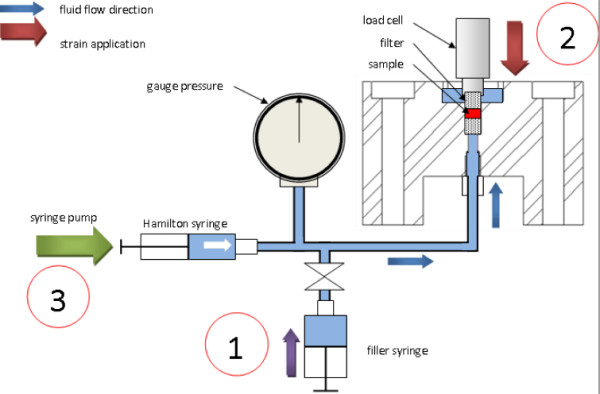
Experimental setup to measure the axial permeability.

The apparatus used to measure the axial permeability consisted of a cylindrical acrylic chamber (Figure 2). Two filters (diameter: 5mm, pore size: 60μm) allowed a fluid to circulate through the tissue sample. The upper filter was fixed to the actuator controlled electronically by the mechanical testing machine (Mach-1, Biomomentum, Montreal, QC, Canada). The fluid flow was regulated by a syringe pump (Cole Parmer, Vernon Hills, IL) using a 500μl Hamilton syringe and the flow pressure inside the system was measured by a 15psi gauge pressure. The outside pressure was the atmospheric pressure. The system was filled with PBS, the sample was introduced carefully in its chamber and the thickness was immediately measured with a tare load of 1g. The contact was maintained for 1h to obtain the stress equilibrium. The reacting force was continuously monitored. The fluid was injected from the syringe pump at a high rate (400μl/h) until a pressure difference across the sample of 40±0.5kPa was recorded. A low fluid rate was then injected (in a range of 20-25μl/h) and the fluid flow was manually adjusted until the equilibrium was reached, for a slope rate lower than 0.01g/min during a 5 min period. The hydraulic permeability (k_a_) was calculated from the Darcy law: for a laminar fluid flow with a low Reynolds number, the intrinsic permeability k (m^4^/Ns) of a saturated porous sample is derived from Equation 7 where Q (m^3^/s) is the flow rate, ΔP (Pa) is the pressure difference, μ (Ns/m^2^) is the fluid dynamic viscosity, l (m) is the length of the sample and S (m^2^) is the cross-section of the sample [43,44].

(7) $$k=\frac{Ql\mu }{\Delta PS}$$

### Statistical analyses

Only the compressive modulus and the permeabilities were chosen for the statistical tests as they presented the smallest standard deviation per group and represent the most important mechanical properties of a biphasic material. For each mechanical property or MRI parameter, a one way ANOVA was performed on the 4 groups followed by a post-hoc multiple comparison with the Dunn-Sidak method. Multi linear regressions were performed between dependent (E, k_r_, H_A0_, k_0_ and k_a_) and independent (T1, T2, MT, FA and ADC) variables to verify our hypothesis.

However, the MR parameters might be found dependant. Thus, a principal component analysis was used to convert the set of possibly correlated variables into a set of linearly uncorrelated variables. The data were first centered and reduced, and organized as a matrix where each row represents a different observation of the experiment and each column gives a different mechanical or MR parameter. The covariance matrix and its eigenvectors and eigenvalues were computed. The cumulative energy content for each eigenvector was used to select a subset of eigenvectors as basis vectors. The source data were then converted into the new basis. The first principal component (F1) has the largest possible variance, and each succeeding component (F2, F3, …Fn) in turn has the highest variance possible under the constraint that it is orthogonal to the preceding components.

Agglomerative Hierarchical Clustering was performed on the 3 first principal components (F1, F2 and F3) computed from the mechanical properties or the MRI parameters. Each observation was a cluster and the process successively merged clusters into larger clusters until it reached one big cluster containing all the samples. We used the Euclidian distance to determine a pairwise distance metric between each observation. The merging of clusters, or linkage, was based on the calculation of the Ward’s distance between clusters. Ward’s linkage uses the incremental sum of squares; that is, the increase in the total within-cluster sum of squares as a result of joining two clusters. The within-cluster sum of squares is defined as the sum of the squares of the distances between all objects in the cluster and the centroid of the cluster. These successive clustering operations produced a binary clustering tree (dendrogram), whose roots contained all the observations.

All statistical tests were performed using XLSTATS (Addinsoft, New York, United States). All results were expressed as Mean±SD and the significance of all tests was set to p≤0.05.

## Results

The enzyme treatment induced mechanical changes in both the annulus fibrosus and nucleus pulposus, with a decrease of the compressive moduli and an increase of the permeabilities (Table 2). For both the annulus fibrosus and nucleus pulposus, ADC decreased while FA increased between *in-situ* and *digested* groups while there were no changes on T1, T2, and MT (Table 3). The one-way ANOVA confirmed significant differences on the compressive modulus between *in-situ* and *digested* groups, but not between the *digested* groups, and on the radial permeability between the *digested* groups and between the *in-situ* and *digested 24h* groups (Table 4). Significant differences were found on the axial permeabilities between the *in-situ* and *digested* groups, but only for the nucleus pulposus. No significant differences were found on T1 and MTR between all groups. Significant differences were found on T2 for the annulus fibrosus, on ADC for the nucleus pulposus and on FA for both annulus fibrosus and nucleus pulposus.

**Table 2:** **Young’s modulus E (MPa), compressive modulus H**_**A0 **_**(MPa), permeabilities k**_**r**_**, k**_**0 **_**and k**_**a **_**(e**^**-15**^**m**^**4**^**/Ns) presented as mean ± SD for all groups (n=15 per group)**

	**E**	**k**_**r**_	**H**_**A0**_	**k**_**0**_	**k**_**a**_
***Nucleus pulposus***					
**In-situ**	0.019±0.017	32±27	0.12±0.08	15±17	10±2
**Dig 6h**	0.008±0.007	74±40	0.04±0.04	31±15	13±3
**Dig 18h**	0.012±0.015	111±154	0.03±0.03	26±19	14±2
**Dig 24h**	0.007±0.008	218±210	0.02±0.01	40±22	16±3
***Annulus fibrosus***					
**In-situ**	0.035±0.030	11±8	0.22±0.17	20±27	8±4
**Dig 6h**	0.029±0.019	12±17	0.09±0.06	11±16	7±2
**Dig 18h**	0.023±0.018	21±16	0.04±0.03	37±28	7±2
**Dig 24h**	0.012±0.013	59±58	0.07±0.06	9±8	9±2

**Table 3:** **Relaxation times T1 and T2 (ms), magnetization transfer ratio MTR (x100) and diffusion parameters FA and ADC (mm**^**2**^**/s) presented as mean ± SD for all groups (n=15 per group)**

	**T1**	**T2**	**MTR**	**FA.10**^**-2**^	**ADC.10**^**-4**^
***Nucleus pulposus***					
**In-situ**	1140±76	124±16	34±20	8.03±4.59	15.04±0.62
**Dig 6h**	1056±130	87±26	28±15	18.59±6.67	15.25±1.58
**Dig 18h**	1115±130	123±5	33±24	8.15±4.52	14.79±1.13
**Dig 24h**	1144±78	118±7	32±22	13.03±7.08	14.17±0.84
***Annulus fibrosus***					
**In-situ**	706±44	70±11	44±15	15.83±3.05	15.89±1.06
**Dig 6h**	659±71	62±11	38±13	23.87±5.24	16.87±0.95
**Dig 18h**	663±73	65±5	42±18	17.10±4.60	15.34±1.27
**Dig 24h**	710±85	68±5	42±16	20.89±4.93	15.42±1.13

**Table 4:** p-values from the one way ANOVA performed on the 4 groups for each mechanical property or MRI parameter of the nucleus pulposus and annulus fibrosus

	**E**	**k**_**r**_	**H**_**A0**_	**K**_**0**_	**k**_**a**_	**T1**	**T2**	**MTR**	**ADC**	**FA**
***Nuclus Pulposus***										
**Global**	0.09	0.002	0.0001	0.03	0.0001	0.19	0.0003	0.88	0.10	0.0002
**In situ / Dig. 6h**	0.07	0.53	0.0002	0.07	0.006	0.06	0.0001	0.45	0.65	0.0001
**In situ / Dig. 18h**	0.73	0.66	0.0001	0.2	0.0001	0.58	0.94	0.89	0.58	0.96
**In situ / Dig. 24h**	0.03	0.001	0.0001	0.004	0.0001	0.92	0.53	0.75	0.06	0.04
**Dig. 6h / Dig. 18h**	0.15	0.85	0.67	0.58	0.14	0.20	0.0002	0.54	0.32	0.0001
**Dig. 6h / Dig. 24h**	0.77	0.0006	0.36	0.26	0.01	0.05	0.001	0.66	0,02	0.03
**Dig. 18h / Dig. 24h**	0.08	0.003	0.64	0.09	0.30	0.52	0.58	0.86	0.18	0.05
***Annulus Fibrosus***										
**Global**	0.04	0.002	0.001	0.05	0.38	0.18	0.33	0.86	0.008	0.0004
**In situ / Dig. 6h**	0.52	0.84	0.005	0.40	0.43	0.12	0.009	0.40	0.04	0.0001
**In situ / Dig. 18h**	0.06	0.44	0.0001	0.14	0.23	0.16	0.26	0.82	0.24	0.5
**In situ / Dig. 24h**	0.01	0.001	0.001	0.24	0.71	0.88	0.67	0.78	0.31	0.009
**Dig. 6h / Dig. 18h**	0.22	0.57	0.21	0.02	0.67	0.88	0.55	0.55	0.002	0.001
**Dig. 6h / Dig. 24h**	0.05	0.001	0.5	0.75	0.25	0.09	0.19	0.57	0.003	0.12
**Dig. 18h / Dig. 24h**	0.47	0.007	0.53	0.01	0.12	0.12	0.49	0.96	0.87	0.05

Multi linear regressions showed that 52 to 70% of E and H_A0_ can be explained by the MRI parameters within the annulus fibrosus, except for E within the *digested 6h* group and for H_A0_ within the *in-situ* group (Table 5). Within the nucleus pulposus, 45 to 68% of E and H_A0_ can be explained by the MRI parameters, except for H_A0_ within the *digested 6h* and *digested 24h* groups. 52 to 80% of the radial permeability k_r_ can be explained by the MRI parameters for both annulus fibrosus and nucleus pulposus, except for the annulus fibrosus within the *in-situ* group. 32 to 65% of the axial permeability in absence of deformation k_0_ and 41 to 70% of the axial permeability k_a_ can be explained by the MRI parameters.

**Table 5:** **Coefficient of determination R**^**2**^**, standard error of estimate (MPa for E and H**_**A0**_**, e**^**-15**^**m**^**4**^**/Ns for k**_**r**_**, k**_**0 **_**and k**_**a**_**) and power of the performed test with α=0.05 of the multilinear regressions between the mechanical properties and the MRI parameters for the nucleus pulposus and the annulus fibrosus**

**Coefficient of determination R**^**2 **^**Standard error of estimate Power with α=0.05**	**E**	**k**_**r**_	**H**_**A0**_	**k**_**0**_	**k**_**a**_
***Nucleus Pulposus***					
*In-situ*	0.48	0.53	0.48	0.49	**0.70**
	0.04	55.6	0.08	73.4	1.2
	0.73	0.79	0.72	0.74	0.95
*Digested-6h*	0.48	**0.62**	0.34	0.45	**0.67**
	0.007	34.6	0.06	15.6	2.2
	0.67	0.85	0.47	0.63	0.90
*Digested-18h*	**0.68**	**0.62**	0.45	0.34	0.55
	0.01	151.9	0.05	21.9	2.1
	0.91	0.85	0.63	0.48	0.76
*Digested-24h*	**0.65**	**0.63**	0.27	0.38	0.41
	0.006	173.7	0.03	24.5	3.2
	0.91	0.90	0.41	0.58	0.63
***Annulus Fibrosus***					
*In-situ*	0.52	0.17	**0.65**	**0.65**	0.48
	0.03	9.8	0.14	40.4	3.6
	0.78	0.26	0.91	0.88	0.73
*Digested-6h*	0.15	0.52	0.49	0.39	0.45
	0.02	41.7	0.06	62.5	2.1
	0.21	0.73	0.72	0.49	0.63
*Digested-18h*	**0.70**	**0.66**	**0.63**	0.32	0.48
	0.01	13.1	0.03	266.2	2.2
	0.92	0.89	0.86	0.44	0.68
*Digested-24h*	0.01	**0.80**	0.21	0.32	**0.71**
	0.93	35.1	0.07	34.1	1.6
	**0.67**	0.99	0.32	0.48	0.95

The linear regressions found for each mechanical property included all the MRI parameters measured in this study (Equation 8). The variance inflation factor (VIF) of T1 and T2 was always higher than the VIF of MT, ADC and FA, suggesting that these parameters were likely candidates for elimination in the equation. However, after removing T2 that presented the highest VIF, all parameters had equivalent VIF and the coefficient of determination did not change significantly.

(8) $$\begin{array}{l} MP=a_{0}+a_{1}\;T1+a_{2}\;T2+a_{3}\;MT\\ \;+a_{4}\;ADC+a_{5}\;FA \end{array}$$

Where MP is one of the mechanical properties E, k_r_, H_A0_, k_0_ and k_a_, and a_i_ (i=0-5) are constants.

**Figure 3:**
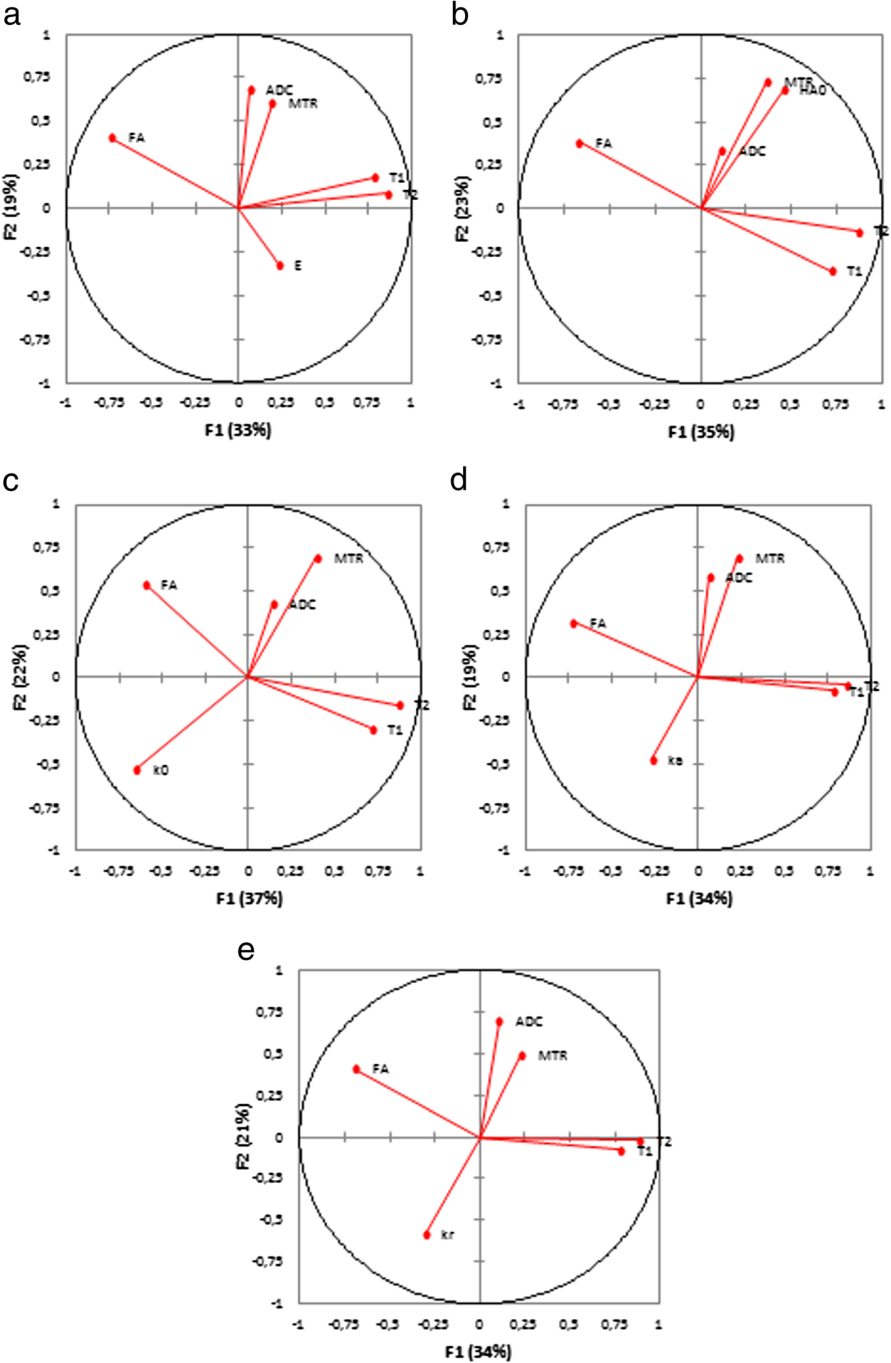
**Principal component analysis, representation of the mechanical property (a- E, b- H**_**A0**_**, c- k**_**0**_**, d- k**_**a**_**, e- k**_**r**_**) and MRI parameters (T1, T2, MTR, ADC and FA) in the (F1, F2) plane for the nucleus pulposus.**

**Figure 4:**
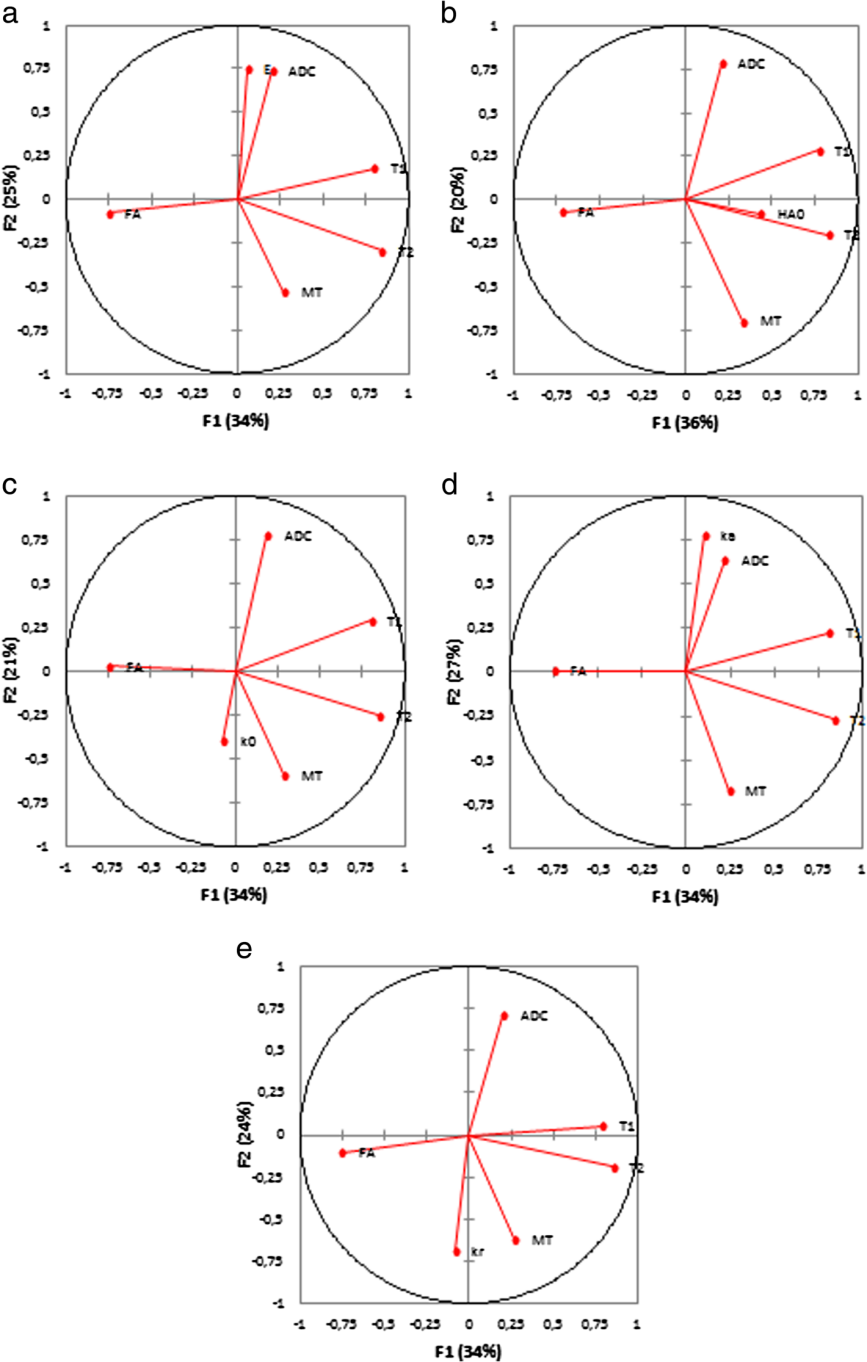
**Principal component analysis, representation of the mechanical property (a- E, b- H**_**A0**_**, c- k**_**0**_**, d- k**_**a**_**, e- k**_**r**_**) and MRI parameters (T1, T2, MTR, ADC and FA) in the (F1, F2) plane for the annulus fibrosus.**

The principal component analysis reduced our six variables (one mechanical property E, k_r_, H_A0_, k_0_ or k_a_, and five MR parameters T1, T2, MTR, ADC and FA) to two principal components F1 and F2 with a cumulative variability of 52-65%, which increased to 70-82% when considering the third principal component F3. The representation of the six variables in the (F1, F2) plane for the nucleus pulposus (Figure 3) showed correlations between T1 and T2 as they were located near the circle and near to each other. Their position near the X-axis suggested that F1 expressed mainly these parameters. ADC, E, k_a_ and k_r_ were far away from the circle, which suggested that these parameters were not expressed only by F1 or F2. The eigenvectors of the covariance matrix showed that these parameters were expressed more by F3 than F1 or F2. The representation of the six variables in the (F1, F2) plane for the annulus fibrosus (Figure 4) showed no correlations between the parameters, except for T1 and FA that were located near the circle and seems to be symmetric relatively to the circle origin. MT, FA, H_A0_, k_0_ and k_r_ were expressed more by F3 than F1 or F2.

**Figure 5:**
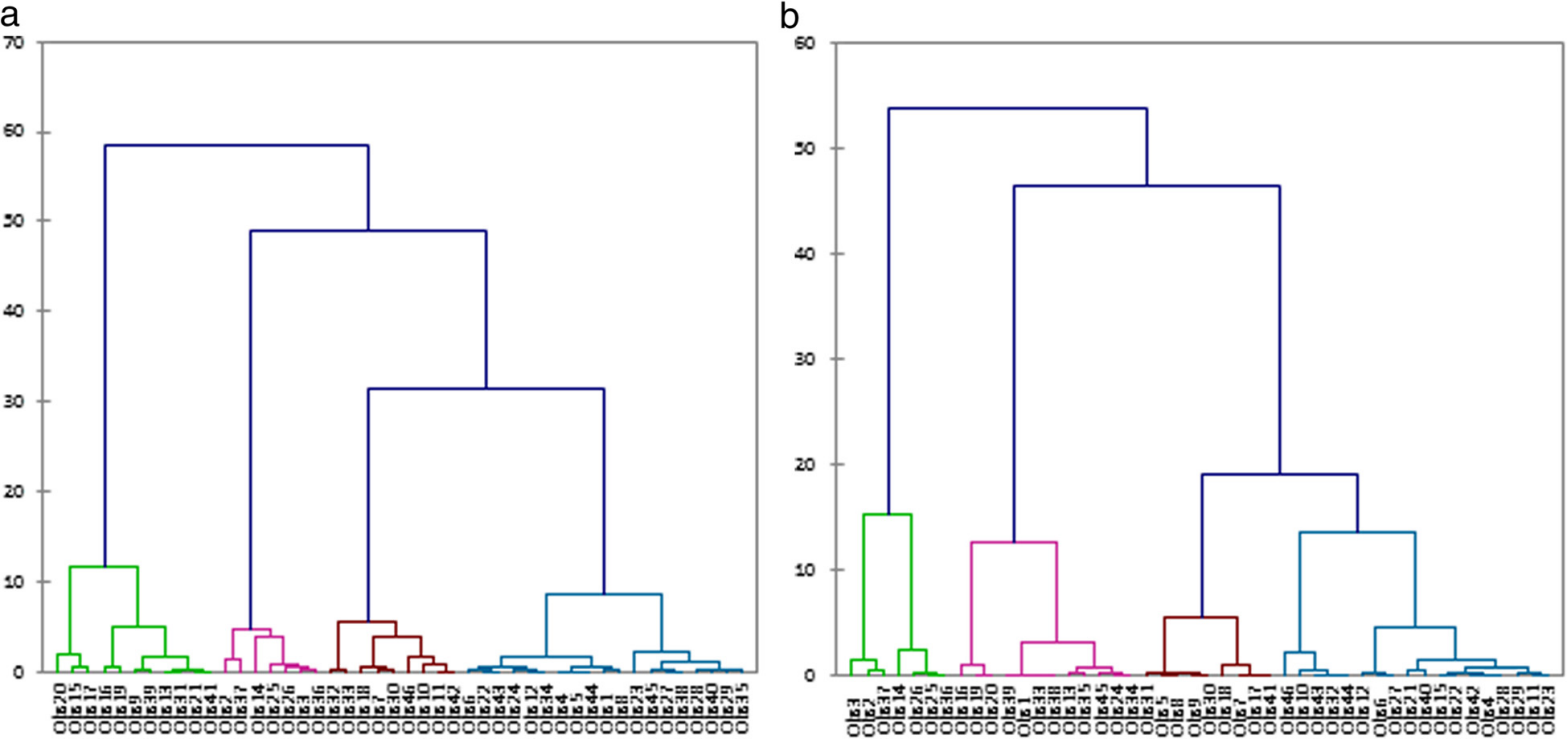
**Dendograms generated from the principal components of the MRI parameters for the nucleus pulposus (a) and the annulus fibrosus (b).** The Y axis is the distance between the two objects being connected and the X axis is the node number.

**Figure 6:**
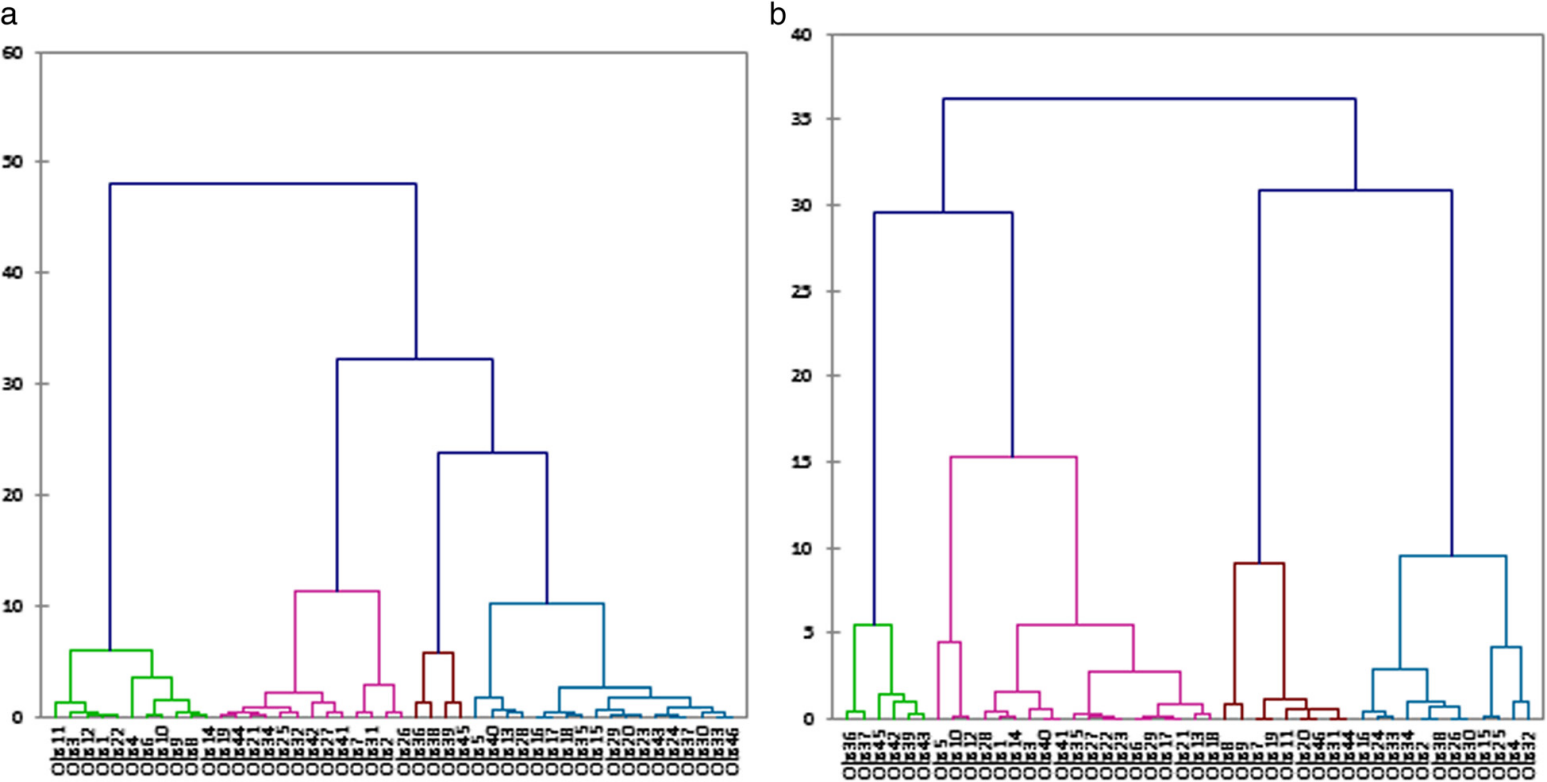
**Dendograms generated from the principal components of the mechanical properties for the nucleus pulposus (a) and the annulus fibrosus (b).** The Y axis is the distance between the two objects being connected and the X axis is the node number.

One way to determine the natural cluster division in a dataset is to compare the height of each link in the dendogram. A link, whose height differs noticeably from the height of the links below, indicates that the objects joined at this level are much farther apart from each other than their components were when they were joined. The dendograms obtained from the MRI parameters showed a natural division into four clusters for the nucleus pulposus but only three clusters for the annulus fibrosus (Figure 5). Indeed, above those four clusters for the nucleus pulposus or three clusters for the annulus fibrosus, no link differs noticeably from the height of the links bellow. The dendograms obtained from the mechanical properties showed a natural division into 4 clusters for both the annulus fibrosus and nucleus pulposus (Figure 6). From the mechanical parameters of the nucleus pulposus, the first cluster contained samples from the *in-situ* group, the second from the *digested 18h* and *digested 24h* groups, the third from the three *digested* groups and the fourth from the *digested 24h* group. From the mechanical properties of the annulus fibrosus, the first cluster contained samples from the *in-situ* and *digested 6h* groups, the second from the *digested 18h* group, the third from the *in-situ* group and the fourth from the *digested 24h* group. From the MRI parameters of both the nucleus pulposus and annulus fibrosus, all the clusters contained samples from all groups.

## Discussion

Multi-parametric MRI acquisitions, unconfined and confined stress-relaxation tests in compression and direct permeability measurements were performed on isolated bovine IVDs, as opposed to previous studies done on bovine tail segments [14,25,26]. Isolated IVDs soaking in a trypsin solution was efficient to simulate degradation as it resulted in an increase of the hydration, a proteoglycan decrease in the nucleus pulposus and an alteration of the structure in both annulus fibrosus and nucleus pulposus. However, this enzyme digestion is not representative of in vivo degeneration mainly because of the water content, which increases with enzyme digestion and decreases with degeneration. However, the aim of this study was to assess the ability of multi-parametric MRI to quantify the mechanical properties of IVDs presenting various compressive modulus or permeabilities.

The mechanical properties were sensitive to our enzyme digestion: the compressive modulus decreased while the permeability increased, in agreement with the literature [23]. The ability of the disc tissue to withstand mechanical forces largely depends on the structural integrity of the matrix and on the biochemical contents [9,45]. The H_A0_ decrease is associated to both structural matrix integrity and biochemical content. The permeability, highly anisotropic for healthy IVDs, becomes slightly more isotropic with degeneration. The degeneration process is associated with a change in water content and induces an increase in the pore size in the radial direction only, but no change in the porosity between collagen lamella [29]. Thus, in this study, the changes in the permeability are dependent on the fluid flow direction, for which the collagen orientation plays an important role, and might also be due to the tissue hydration.

Trypsin digestion did not affect T1, T2 and MTR, as previously reported on bovine tails [14,25]. T1 has been shown to correlate with disc water content and not with proteoglycan content. T2 has been shown to be an indicator for the integrity of the collagen network, and MTR is known to be correlated to the collagen structure and composition [18], and thus our digestion process seems to have not affected the collagen structure or content. This is justified knowing that trypsin catalyzes the hydrolysis of peptide bonds at the carboxyl side of lysyl and arginyl residues [46], and thus principally cleaves the proteoglycan core protein [14]. The most sensitive MRI parameters were ADC and FA. The decrease of ADC reflects less diffusion within the tissue, which might be associated to less nutrition in vivo [12], and consequently to IVD degradation. The increase of FA reflects a structural integrity that entails a preferred direction of the fluid flow within the tissue. We previously demonstrated that, while the trypsin digestion increased the anisotropy, the hydration decreased the anisotropy and thus increased the disorganization of the collagen fibers within the annulus fibrosus. Significant differences were found only between the *in situ* and *digested 6h* groups for T2. In this *digested 6h* group, there is the effect of the hydration and not of the enzyme digestion. We previously demonstrated that a progressive decrease of both T1 and T2 between *in-situ* and hydrated groups, and a progressive increase between the hydrated and digested groups were observed. The effect of proteoglycans cleaving from trypsin digestion on the MRI relaxation times is hidden by the effect of hydration. The changes in the MRI parameters are smaller than the changes in the mechanical properties. The MR relaxation times are not the best candidates to reflect the changes in the mechanical properties. However, the diffusion parameters are sensitive to these changes.

The MRI parameters chosen in this study (T1, T2, MT, FA, ADC) can assess the compressive moduli and the radial or axial permeability, in agreement with the literature [11,25]. However, our results suggest that the relaxation time T2 could be removed from the equations because of its high variance inflation factor. T1ρ, which is known to be more sensitive to the IVD degenerescence than T1 and T2 [14,25], would be the perfect candidate to improve our regressions. The expression of the multi-linear regressions between the mechanical properties and MRI parameters differs from a group to another one, for both annulus fibrosus and nucleus pulposus. The differences between the *digested* groups might be due to the method used to quantify the mechanical properties of the IVD. While we achieved the highest possible consistency of protocol in the mechanical testing, there were technical challenges in measuring nucleus pulposus tissues due to the extremely low stiffness and lack of geometric stability, particularly for the enzymatically treated samples. This point is illustrated by the high standard deviation found for k_0_. However, the direct measurement of the permeability reduces the standard deviations and the differences between the linear regressions. The quantification of the MR parameters is more accurate than the determination of the mechanical properties because the MRI protocol was rigorously the same for all acquisitions and the annulus fibrosus or nucleus pulposus segmentations were fully automatic using a Canny edge detection algorithm. The differences between the *in-situ* and *digested* groups might be due to the sensitivity of the MR parameter to a specific change in the composition or structure of the tissue. Thus, the linear regressions between the mechanical properties and MRI parameters have to be defined for each type of degeneration that mimics the pathology we want to study in vivo.

The reduction of the variables to two or three principal components confirmed that the relationships between the mechanical properties and MRI parameters may be non linear. Principal component analysis is very useful to reduce the dimensionality of a data set by projecting high dimensional data into a lower dimensional space. The natural division into three or four clusters on the dendrograms from the MRI parameters did not reflect our 4 experimental groups, due to the lack of differences between groups for T1, MTR and ADC. The natural division into four clusters on the dendograms from the mechanical properties reflected our 4 experimental groups. However, the differentiation between the *in-situ* and the *digested 6h* groups was not clear for the annulus fibrosus. The mechanical properties were more able to classify the degenerated IVDs than the MRI parameters.

Only 15 samples were considered per group. The power of all the significant statistical tests was over 0.8, justifying this number of samples. The quantification of the mechanical properties was limited to compression tests. The compression loading on the intervertebral disc in vivo is expected to result in large hydrostatic pressures within the nucleus pulposus. Thus the relationships established between the mechanical properties and MRI parameters are appropriated for the nucleus pulposus. However, the highly oriented annulus fibrosus is submitted to more complicated deformation patterns. Shear is an important loading mode in the annulus fibrosus, particularly relevant under bending and torsion loading of the IVD. Future studies of the relationships between mechanical properties and MRI parameters within the annulus fibrosus should include shear tests as well as traction tests. Diffusion tensor imaging might be the most relevant MRI technique to reflect the shear behavior of the tissue.

## Conclusions

Multi-parametric MRI is a sensitive and non-invasive technique for describing the alterations in mechanical properties of IVDs. This study showed that the compressive modulus and the permeability of isolated IVDs can be assessed mostly by magnetization transfer sequences and diffusion tensor imaging. However, the relationships have to be improved with the inclusion of MRI parameters more sensitive to IVD degeneration such as T1ρ or CEST (Chemical Exchange Saturation Transfer). Before the use of this technique to quantify the mechanical properties of IVDs in vivo on patients suffering from various diseases, the relationships have to be defined for each degeneration type of the tissue that mimics the pathology. Our MRI protocol associated to principal component analysis and agglomerative hierarchical clustering are promising tools to classify the degenerated intervertebral discs and further find biomarkers and predictive factors of the evolution of the pathologies.

## Competing interests

The authors declare that they have no competing interests, except GG who receives financial support (salary) from Philips Healthcare.

## Authors’ contributions

MR carried out the experiments, all the data analysis, discussed the results and drafted the manuscript. DP proposed the design of the study, participated in the results discussion and helped to draft the manuscript. GG carried out the MRI experiments, participated to the data analysis and to the results discussion, and corrected the manuscript. GB participated in the results discussion and corrected the manuscript. All authors read and approved the final manuscript.

## Pre-publication history

The pre-publication history for this paper can be accessed here:

http://www.biomedcentral.com/1471-2474/13/195/prepub
